# Effects of immune checkpoint inhibitor associated endocrinopathies on cancer survival

**DOI:** 10.3389/fendo.2024.1369268

**Published:** 2024-04-12

**Authors:** Lisa Yang, Sruthi Murthy, Alessio Cortellini, Emma A. Lim, Michael Gonzalez, David J. Pinato, Mariana Abdel-Malek, Sarah Mahmoud, Niamh M. Martin

**Affiliations:** ^1^ Department of Endocrinology, Imperial College Healthcare NHS Trust, London, United Kingdom; ^2^ Department of Surgery & Cancer, Imperial College London, London, London, United Kingdom; ^3^ Operative Research Unit of Medical Oncology, Fondazione Policlinico Universitario Campus Bio-Medico, Rome, Italy; ^4^ Department of Imaging, Imperial College Healthcare NHS Trust, London, United Kingdom; ^5^ Department of Medical Oncology, Imperial College Healthcare NHS Trust, London, United Kingdom; ^6^ Division of Oncology, Department of Translational Medicine, University of Piemonte Orientale, Novara, Italy; ^7^ Department of Pharmacy, Imperial College Healthcare NHS Trust, London, United Kingdom; ^8^ Section of Endocrinology and Investigative Medicine, Division of Diabetes, Endocrinology and Metabolism, Department of Metabolism, Digestion and Reproduction, Imperial College London, London, United Kingdom

**Keywords:** immune checkpoint inhibitor, endocrinopathy, cancer, survival, immune related adverse effects (irAEs)

## Abstract

**Objectives:**

Immune checkpoint inhibitors (ICIs) are associated with immune-related adverse events (irAEs), of which endocrinopathies are common. We characterized endocrine and non-endocrine irAEs in cancer patients receiving ICIs, identified risk factors for their development and established whether endocrine and non-endocrine irAEs were differentially associated with improved cancer prognosis.

**Design and methods:**

Single-center, retrospective cohort study of patients with advanced or metastatic solid tumors receiving at least one ICI treatment cycle (242 men, 151 women, median age 65 years). Main outcome measures were incidence of any irAE during the study period, overall survival and time to treatment failure.

**Results:**

Non-endocrine irAEs occurred in 32% and endocrine irAEs in 12% of patients. Primary thyroid dysfunction was the most common endocrine irAE (9.5%) and the majority of endocrinopathies required permanent hormone replacement. Women had an increased risk of developing endocrine irAEs (p = 0.017). The biggest survival advantage occurred in patients who developed both endocrine and non-endocrine irAEs (overall survival: HR 0.16, CI 0.09-0.28). Time to treatment failure was also significantly improved in patients who developed endocrine irAEs (HR 0.49, CI 0.34 – 0.71) or both (HR 0.41, CI 0.25 – 0.64) but not in those who only developed non-endocrine irAEs.

**Conclusions:**

Women may have increased risk of endocrine irAEs secondary to ICI treatment. This is the first study to compare the effects of endocrine irAEs with non-endocrine irAEs on survival. Development of endocrine irAEs may confer survival benefit in ICI treatment and future, prospective studies are needed to elucidate this.

## Introduction

Immune checkpoint inhibitors (ICIs) upregulate the adaptive immune system by promoting T-lymphocyte mediated cytotoxic cell-death. In doing so, ICIs have revolutionised cancer care with excellent response rates and improved overall survival in multiple cancer types (1). More recently, it has been proposed that ICIs may also have a potential therapeutic role in more rare tumor subtypes such as phaeochromocytoma and paraganglioma (2).

ICIs are classified into three groups: cytotoxic T-lymphocyte-associated protein 4 (CTLA-4), programmed cell death protein 1 (PD-1) and programmed death-ligand 1 (PD-L1) inhibitors (1). Ipilimumab was the first ICI to be approved and remains a commonly used CTLA-4 inhibitor, targeting CTLA-4 on CD4 positive and CD8 positive T lymphocytes. This was swiftly followed by anti-PD-1 therapies, including nivolumab and pembrolizumab, targeting PD-1 on CD8 positive T lymphocytes. More novel anti-PD-L1 therapies, including atezolizumab, avelumab and durvalumab, bind directly to PD-L1 expressed on the tumor itself (3).

While activation of the immune system by ICIs provides significant therapeutic benefits, these drugs are also associated with immune-related adverse events (irAEs) which can be challenging to predict, diagnose and treat (3). IrAEs typically occur in a dose-dependent fashion with regimens containing CTLA-4 inhibitors, but are less predictable for PD-1 and PD-L1 inhibitors administered as monotherapies (4, 5). IrAEs can occur in any organ system, but more frequently occur in those with extensive environmental interfaces (e.g., skin, lungs, liver and gastrointestinal tract) or those with increased predisposition to autoimmunity (e.g., thyroid and joints). In general, irAEs occur most often during the first three months of treatment but can arise at any time during therapy and after treatment cessation (6).

Endocrine irAEs are among the most commonly encountered irAEs in patients receiving ICI therapy with a reported incidence of 15 – 40% depending on ICI type (5, 7). The most common endocrine irAEs encompass thyroid dysfunction, primary adrenal insufficiency, hypophysitis and immune-mediated diabetes mellitus (5). As with other irAEs, onset is more common during the first few months of treatment, but can also occur at any time during and after treatment (5). Endocrine irAEs often remain undiagnosed for some time due to the non-specific manifestations of endocrinopathies which can overlap with cancer-related or therapy-related complications (e.g., fatigue, loss of libido, depression, nausea) (5). Although often considered mild compared to other irAEs, endocrine irAEs can present as emergencies and fatalities have been reported, highlighting the need for prompt recognition and treatment (8, 9).

A growing body of evidence suggests that development of irAEs is associated with improvements in progression-free survival, overall survival and overall disease response rate (10–13). Studies have demonstrated shared T cell receptor sequences and/or upregulated organ-specific transcripts between tumors and non-malignant tissues affected by irAEs, which suggest that irAEs and the anti-tumor immune response to ICIs could be mechanistically linked (14). Prior studies investigating survival outcomes and irAEs have been focussed on clinical trial data and although valuable, can be limited by small numbers, single agent use, or disease-site specific studies, and may not reflect standard clinical care (15–17). Real-world studies evaluating the incidence and management of endocrine irAEs and assessing their effects on survival, will contribute to establishing best practice within every day clinical care.

In this study, we characterise endocrine and non-endocrine irAEs in cancer patients receiving ICIs within the standard clinical practice of a single tertiary center. We aimed to identify risk factors for development of endocrine irAEs and to establish whether development of endocrine and/or non-endocrine irAEs are associated with improved cancer prognosis.

## Methods

### Study design and participants

A single-center retrospective cohort study was conducted at Imperial College Healthcare NHS Trust (ICHNT) with advanced or metastatic solid tumors receiving one of the following ICIs: atezolizumab, avelumab, durvalumab, nivolumab, pembrolizumab or combination therapy with ipilimumab and nivolumab. Using a pharmacy prescribing database, all adult patients (>18y) diagnosed with a cancer eligible for ICI treatment who had received a minimum of one treatment cycle between February 2017 and September 2021 at our center were identified for inclusion in this study. Demographic and clinical information (including a previous medical history of autoimmune disease) were extracted from electronic patient records. Anonymised data were recorded until treatment ended, death or the datalock of 1^st^ March 2022. Patients were excluded from subsequent analysis only if there were insufficient data at follow up, if ICI treatment was not initiated or if patients died prior to starting ICI treatment.

During the organisation of this audit of non-identifiable data from routine clinical practice, the Health Research Authority online decision tool http://www.hra-decisiontools.org.uk/research/ (18) was used which confirmed that regulatory approval by an NHS Research Ethics Committee (REC) was not required. This study was registered as an audit with our institution (audit number END_023).

Non-endocrine and endocrine irAEs were recorded after the first ICI treatment cycle and at each clinical visit, scheduled prior to each treatment cycle. Due to the reported frequency of ICI-induced thyroid dysfunction, all patients had thyroid function measured before ICI treatment initiation and before each treatment cycle. Patients were monitored for the development of all irAEs (non-endocrine and endocrine) at each clinical visit and these were recorded in the electronic patient records which were subsequently carefully reviewed for this study. Specific endocrine tests (i.e., in addition to thyroid function) e.g., pituitary profile, HbA1c, were performed according to symptoms.

### Definitions

Thyroid irAEs were defined as new thyroid dysfunction developing after ICI treatment and were further subcategorised into primary hypothyroidism, subclinical hypothyroidism, isolated hyperthyroidism or biphasic thyroiditis using specific criteria (19, 20).

• Primary hypothyroidism - Thyroid Stimulating Hormone (TSH) concentration above 10mU/L, irrespective of fT4 concentration.
• Subclinical hypothyroidism – TSH concentration above 4mU/L but below 10mU/L, with associated fT4 within the reference range.
• Isolated hyperthyroidism - TSH concentration below 0.5mU/L with associated fT4 above the upper limit of the reference range that is not followed by a hypothyroid phase.
• Biphasic thyroiditis - Transient thyrotoxicosis defined by a TSH concentration below 0.5mU/L (irrespective of fT4 concentration) followed by a hypothyroid phase defined by a high TSH concentration exceeding 4mU/L.

Reference ranges in our institution are: fT4 9-23 pmol/L, fT3 2.4-6 pmol/L and TSH 0.30-4.20 mu/L.

Hypophysitis was defined as new onset deficiency of one or more pituitary hormones and/or evidence of pituitary gland or stalk enlargement on brain or pituitary MRI without prior glucocorticoid treatment (20, 21). All available neuroimaging studies for each patient were reported by a neuroradiologist at the time of acquisition as part of routine clinical care. For this study, a second, independent neuroradiologist (E.A.L.) re-reviewed this imaging and specifically focused on the presence of radiological features of hypophysitis and associated complications including compression of the anterior visual pathways, features suggestive of an alternative cause for a primary/secondary hypophysitis and exclusion of other structural adenomatous/non-adenomatous pituitary and infundibular abnormalities.

Primary adrenal insufficiency was defined as random cortisol concentration < 100 nmol/L or cortisol concentration < 350 nmol/L on short synACTHen testing (250mcg tetracosactide) with baseline ACTH at least twice the upper limit of the reference range (ng/L) without prior glucocorticoid treatment (21).

Diabetes mellitus was diagnosed in patients presenting with new onset hyperglycaemia with subsequent characterisation including measurement of blood ketones, C-peptide, HbA1c and anti-glutamic acid decarboxylase antibodies (anti-GAD) (22).

### Assay characteristics

Laboratory measurement of serum sodium was via indirect ion-selective electrode methodology (Abbott Architect and Alinity-C). Measurement of prolactin, Thyroid Stimulating Hormone (TSH), free triiodothyronine (free T3) and free thyroxine (free T4) used a two-step immunoassay (IA) using chemiluminescent microparticle IA (CMIA) technology (Abbott Architect and Alinity-C). Measurement of serum cortisol involved a one-step IA using CMIA technology (Abbott Architect and Alinity-C). Measurement of adrenocorticotrophic hormone (ACTH) was performed by a solid-phase, two site sequential chemiluminescent immunometric assay (Immulite 2000, Siemens). A delayed one-step IA using CMIA technology was used to measure oestradiol and testosterone in serum (Abbott Architect and Alinity-C). Serum follicle stimulating hormone (FSH) and luteinising hormone (LH) was measured by a two-step IA using CMIA technology (Abbott Architect and Alinity-C).

C-peptide measurement was carried out using a two-step IA using CMIA technology (Alinity-I analyser, Abbott). Glycated haemoglobin (HbA1c) was measured by ion exchange high performance liquid chromatography (HPLC) (HLC-723G11 analyser, Tosoh) and reported as mmol per mol of unglycated HbA in line with the International Federation for Clinical Chemistry (IFCC) standardisation. Glucose was measured via enzymatic measurement (Hexokinase/Glucose-6-phosphate Dehydrogenase) (Abbott Architect and Alinity-C). Enzyme-linked immunosorbent assay (ELISA) was used to measure thyroid stimulating hormone receptor (TSHR) autoantibody (ElisaRSR TRAb 3^rd^ Generation Kit) as well as glutamic acid decarboxylase (GAD) Autoantibody (ElisaRSR GAdAb Kit), which were tested manually and read using the MRX Dynatec software. The assay for autoantibodies against tyrosine phosphatase-related islet antigen 2 (IA2) was manual ELISA (Euroimmun Kit) and read using the MRX Dynatec software.

### Statistical analysis

Baseline patient characteristics were reported with descriptive statistics as appropriate. The χ2 test was used to compare categorical variables. The Kruskal-Wallis test was used to compare continuous variables.

Oncological outcomes of interest included overall survival (OS), defined as the time from treatment initiation to patient death or loss to follow-up and Time to Treatment Failure (TTF), defined as the time from treatment initiation to its interruption for any cause including death. For OS and TTF, patients without events were considered as censored at the time of the last follow-up. Median OS and TTF were evaluated and compared using the Kaplan-Meier method and the log-rank test according to the experience of endocrine and non-endocrine irAEs. Patients who did not develop any irAEs were set as the control group. Duration of follow-up was calculated according to the reverse Kaplan-Meier method.

A fixed multivariable Cox proportional hazards regression including all the available baseline characteristics (age, sex, primary tumor) was used to estimate the risks of treatment interruption and death, and to compute the hazard ratios (HR) with 95% confidence intervals (CIs). Acknowledging the intrinsic association between the experience of irAEs and treatment exposure, time-adjusted Cox multivariable regression models for the risks of treatment interruption and death were also used.

All P-values were two-sided and confidence intervals set at the 95% level, with significance pre-defined to be at <0.05. Analyses were performed using the MedCalc® Statistical Software version 20 (MedCalc Software Ltd, Ostend, Belgium; https://www.medcalc.org; 2021).

## Results

### Study cohort

452 eligible patients were identified. 54 were excluded (n = 26 insufficient data, n = 19 ICI treatment subsequently contraindicated, n = 9 died prior to starting ICI treatment), leaving 398 patients for inclusion in the study.

Out of the 398 patients included, treatment details were: anti-PD-1 inhibitors: nivolumab (n = 57) or pembrolizumab (n = 201), anti-PD-L1 inhibitors: ateluzumab (n = 85), durvalumab (n = 6) or avelumab (n = 12), combination anti-PD-1 and anti-CTLA4 inhibitors: nivolumab plus ipilimumab (n = 37). For patients receiving concomitant chemotherapy: pembrolizumab; 45 received pemetrexed plus carboplatin, 4 received pemetrexed plus cisplatin, 1 received pemetrexed plus cisoplatin/carboplatin, 8 received paclitaxel plus carboplatin, ateluzumab; 14 received etoposide plus carboplatin, 4 received bevacizumab, carboplatin and paclitaxel, 14 received bevacizumab, 4 received paclitaxel albumin, avelumab; 9 received axitinib.

Baseline patient characteristics are presented in **Table 1**. The study population had a median age of 65 years, 14.8% had a prior history of autoimmunity, and 37.9% were females. Median follow-up was 26.2 months (95%CI: 21.1-30.4). The majority of patients receiving ICI treatment were diagnosed with non small cell lung cancer (NSCLC) (52.5%). Overall, non-endocrine irAEs occurred in 32% (n = 127) and endocrine irAEs occurred in 12% (n = 48) of patients treated with ICIs. 4.0% (n = 16) developed both non-endocrine and endocrine irAEs during ICI treatment.

**Table 1:** Baseline characteristics.

	Overall population (398 patients) N (%)	No irAEs (239 patients) N (%)	Non-endocrine irAEs (127 patients) N (%)	P value	Endocrine irAEs (48 patients) N (%)	P value
**Age** Median (range)	65 (27-100)	64 (27-100)	65 (31-82)	0.374	66 (32-81)	0.854
**Sex** Female Male	151 (37.9) 247 (62.1)	90 (37.7) 149 (62.3)	45 (35.4) 82 (64.6)	0.675	27 (56.2) 21 (43.7)	0.017
**Pre-existing autoimmunity** No Yes	339 (85.2) 59 (14.8)	201 (84.1) 38 (15.9)	110 (86.6) 17 (13.4)	0.903	42 (87.5) 6 (12.5)	0.698
**Primary tumor** NSCLC Melanoma Renal Cell Carcinoma Urothelial Carcinoma HCC Head and Neck Cancers Others	209 (52.5) 42 (10.6) 49 (12.3) 29 (7.3) 14 (3.5) 39 (9.8) 16 (4.0)	129 (54.0) 11 (4.6) 25 (10.6) 23 (9.6) 9 (3.8) 31 (13.0) 11 (4.6)	66 (52.0) 26 (20.5) 20 (15.7) 4 (3.1) 5 (3.9) 3 (2.4) 3 (2.4)	<0.001	22 (45.8) 9 (18.8) 7 (14.6) 2 (4.2) - 5 (10.4) 3 (6.2)	0.013

Continuous parameters are shown as mean ± SD, and categorical variables as n (%) of the overall population. The Kruskal-Wallis test was used to compare continuous variables. The χ2 test was used to compare categorical variables.

NSCLC, non small cell lung cancer; HCC, hepatocellular carcinoma.

### Non-endocrine irAEs

The most common non-endocrine irAE was dermatitis occurring in 10.3% (n = 41) of patients. This was followed by hepatitis which was found in 9.6% (n = 38). Other non-endocrine irAEs encountered in this cohort included inflammatory arthritis, mucositis, pneumonitis, colitis, nephritis and peripheral neuropathy. Rare irAEs (less than two cases) included discitis, glossitis, myositis, sinusitis and vulvitis.

### Endocrine irAEs

#### Primary thyroid dysfunction

Primary thyroid dysfunction was the most frequently encountered endocrinopathy, comprising two thirds of all endocrine irAEs (incidence 9.5%) (**Table 2**). Thyroiditis was the most common cause of primary thyroid dysfunction (22 of 38 cases), occurred earliest (median onset six weeks) and was associated with all classes of ICI, although 50% of cases occurred with the PD-1 inhibitor pembrolizumab. Primary hypothyroidism was associated with anti-PD-1 inhibitors; pembrolizumab and nivolumab and with combination anti-PD-1 and CTLA4 inhibitor therapy (nivolumab plus ipilimumab). Isolated hyperthyroidism (i.e., without subsequent hypothyroidism) presented later than biphasic thyroiditis and primary hypothyroidism, and was associated with anti-PD-1 and anti-PDL1 inhibitors (avelumab, pembrolizumab and durvalumab) but not with combination therapy. All patients who developed biphasic thyroiditis and primary hypothyroidism required long-term treatment with levothyroxine.

**Table 2:** Incidence of endocrine immune-related adverse events (irAEs).

Endocrine irAE	Incidence N (%)	Associated ICI (N)	Time to onset of endocrine irAE in weeks median (range)	Associated non-endocrine irAEs (N)
**Thyroid dysfunction** ** Isolated hyperthyroidism** **Primary hypothyroidism** **Subclinical hypothyroidism** ** Biphasic thyroiditis**	38 (9.5) 3 (0.8) 11 (2.8) 2 (0.5) 22 (5.5)	Avelumab (1) Pembrolizumab (1) Durvalumab (1) Pembrolizumab (6) Nivolumab (4) Combination therapy* (1) Pembrolizumab (2) Pembrolizumab (11) Atezolizumab (7) Combination therapy* (2) Avelumab (1) Nivolumab (1)	10 (3.0 - 12) 8 (3.0 – 27) 21 (15 - 27) 6 (3.0 - 39)	Hepatitis (1) Pneumonitis (1) Pneumonitis (1) Dermatitis (1) None Hepatitis (1) Pneumonitis (1) Dermatitis (5) Mucositis (2) Sinusitis (1)
**Hypophysitis**	9 (2.3)	Combination therapy* (4) Nivolumab (2) Pembrolizumab (3)	9 (4.0 – 24)	Hepatitis (3) Colitis (1) – all occurred with combination therapy
**Diabetes Mellitus**	3 (0.8)	Pembrolizumab (3)	36 (9 – 48)	None
**>1 endocrinopathy**	2 (0.5)	Pembrolizumab (2)	26 (6 – 36)	None

50 endocrine irAEs occurred in 48 patients. Incidence reported as N (%) of the overall population. Categorical variables are reported as median (range). Two patients developed > 1 endocrinopathy; subclinical hypothyroidism and isolated ACTH deficiency occurred in one patient, thyroiditis and type 1 diabetes occurred in the second patient. *Combination therapy with ipilimumab and nivolumab.

### Hypophysitis

Hypophysitis occurred in nine patients (incidence 2.3%), with a median onset of nine weeks (**Table 2**). Two patients presented with headache accompanied by changes consistent with hypophysitis on imaging and multiple pituitary hormone deficiencies both of which received combination anti-PD-1 and CTLA4 inhibitor therapy (nivolumab plus ipilimumab). These multiple pituitary hormone deficiencies were secondary hypoadrenalism (both patients, permanent), secondary hypogonadism (in one patient, transient) and secondary hypothyroidism (both patients, permanent in one, transient in one). Seven patients had isolated ACTH deficiency (three received pembrolizumab, two received nivolumab, two received nivolumab plus ipilimumab), with 5/7 having no appearance of hypophysitis on imaging (two patients did not have imaging). In all nine cases, ACTH deficiency was permanent. AVP deficiency did not occur in any patient.

### Primary adrenal insufficiency

There were no cases of primary adrenal insufficiency identified in this cohort.

### Diabetes mellitus

Diabetes mellitus occurred in three patients (incidence 0.8%), who all received the anti-PD-1 inhibitor pembrolizumab, and presented later than other endocrinopathies (median onset 36 weeks) (**Table 2**). All patients presented with diabetic ketoacidosis and required lifelong insulin therapy. C-peptide levels were low in all three cases (< 27 – 81 pmol/L). Anti-GAD and anti-islet cell antibodies were checked, with only one patient showing positive antibodies.

### Factors associated with development of irAEs

There was no significant difference in age or previous history of autoimmunity between the patients who developed non-endocrine irAEs, endocrine irAEs and those who developed no irAEs. Women had an increased risk of developing endocrine irAEs (p = 0.017) (**Table 1**).

### Overall survival and time to treatment failure

**Figure 1:**
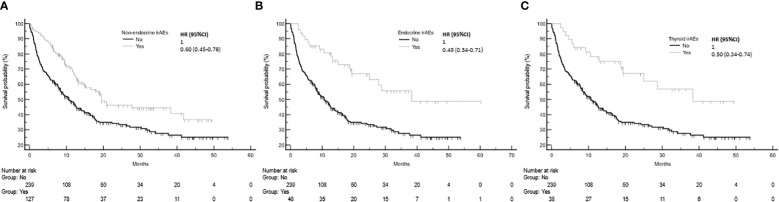
Overall survival in patients with and without non-endocrine, endocrine or thyroid immune related adverse events (irAEs). **(A)** Overall survival among patients who experienced non-endocrine irAEs was 19.5 months (95%CI: 15.1-41.7, 60 events), that of patients who did not experienced irAEs was 10.6 months (95%CI: 8.3-13.9, 152 events), Log-rank p value = 0.001. **(B)** Overall survival of patients who experienced endocrine irAEs was 38.3 months (95%CI: 24.9-38.3, 19 events). Log-rank p value = 0.001 (comparison with no irAEs). **(C)** Overall survival of patients who experienced thyroid irAEs was 38.3 months.

**Figure 2:**
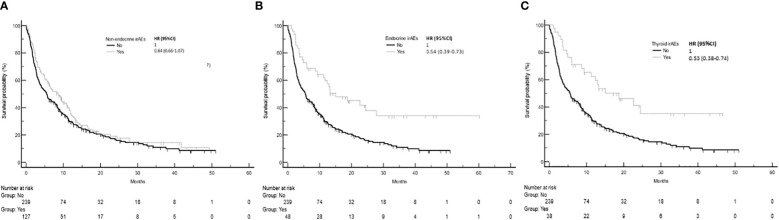
Time to treatment failure in patients with and without non-endocrine, endocrine or thyroid immune related adverse events (irAEs). **(A)** Time to treatment failure among patients who experienced non-endocrine irAEs was 8.6 months (95%CI: 5.7-11.1, 100 events), while that of patients who did not experience irAEs was 5.5 months (95%CI: 3.9-7.5, 192 events), Log-rank p value = 0.154. **(B)** Time to treatment failure of patients who experienced endocrine irAEs was 13.3 months (95%CI: 9.3-24.5, 31 events). Log-rank p value = 0.001 (comparison with no irAEs). **(C)** Time to treatment failure of patients who experienced thyroid irAEs was 15.0 months (95%CI: 9.3-24.4, 23 events). Log-rank p values = 0.001 (comparison with no irAEs).

**Figure 3:**
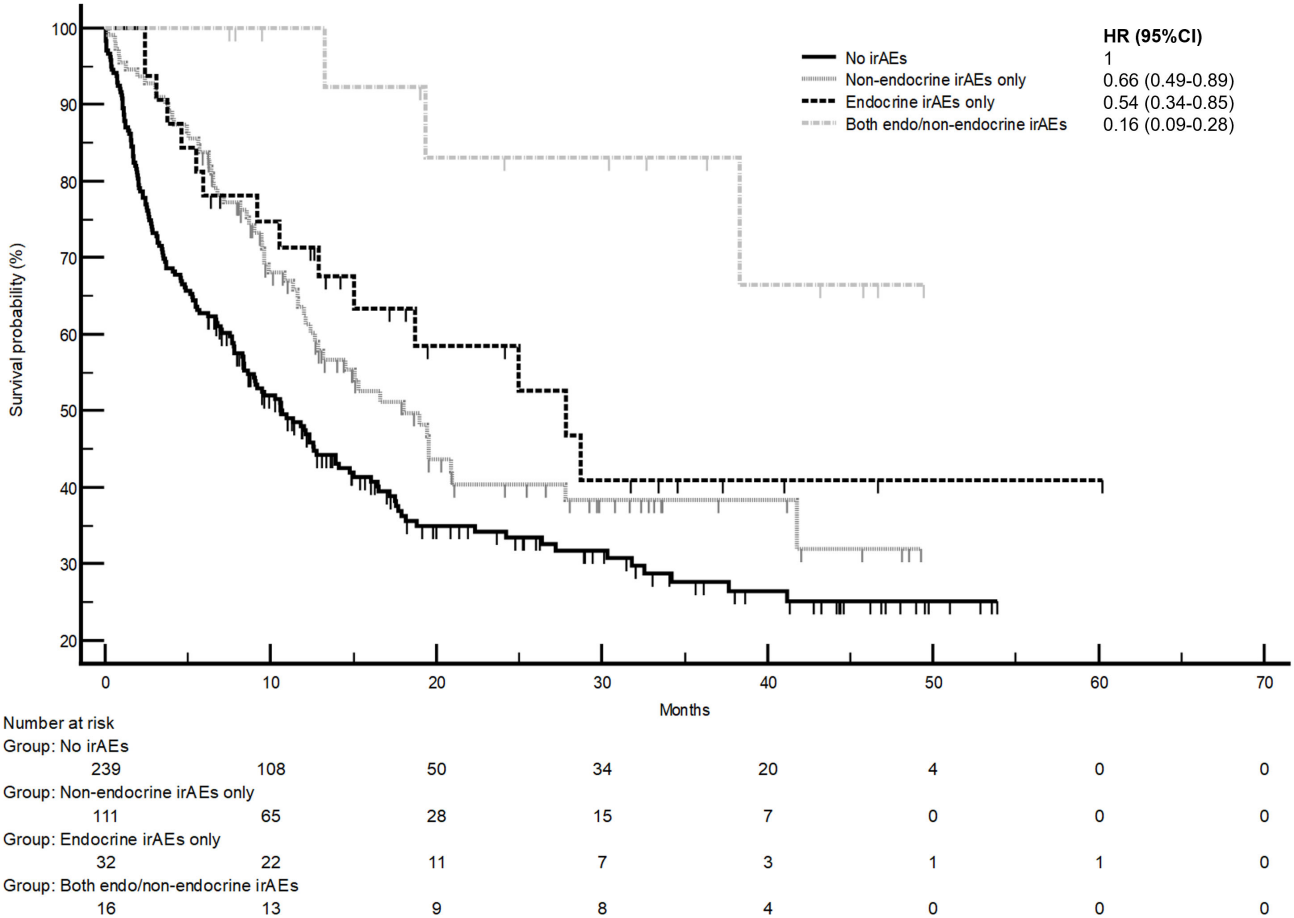
Comparison of overall survival in patients with and without endocrine and/or non-endocrine immune related adverse events (irAEs). Overall survival of patients who experienced non-endocrine irAEs only was 18.0 months (95%CI: 12.4-27.8, 57 events), that of patients who experienced endocrine irAEs only was 27.8 months (95%CI: 12.9-27.7, 15 events), that of patients who experienced both non-endocrine and endocrine irAEs was not reached (three events). Log-rank p values = 0.001 (comparison with no irAEs for hazard ratio).

**Figure 4:**
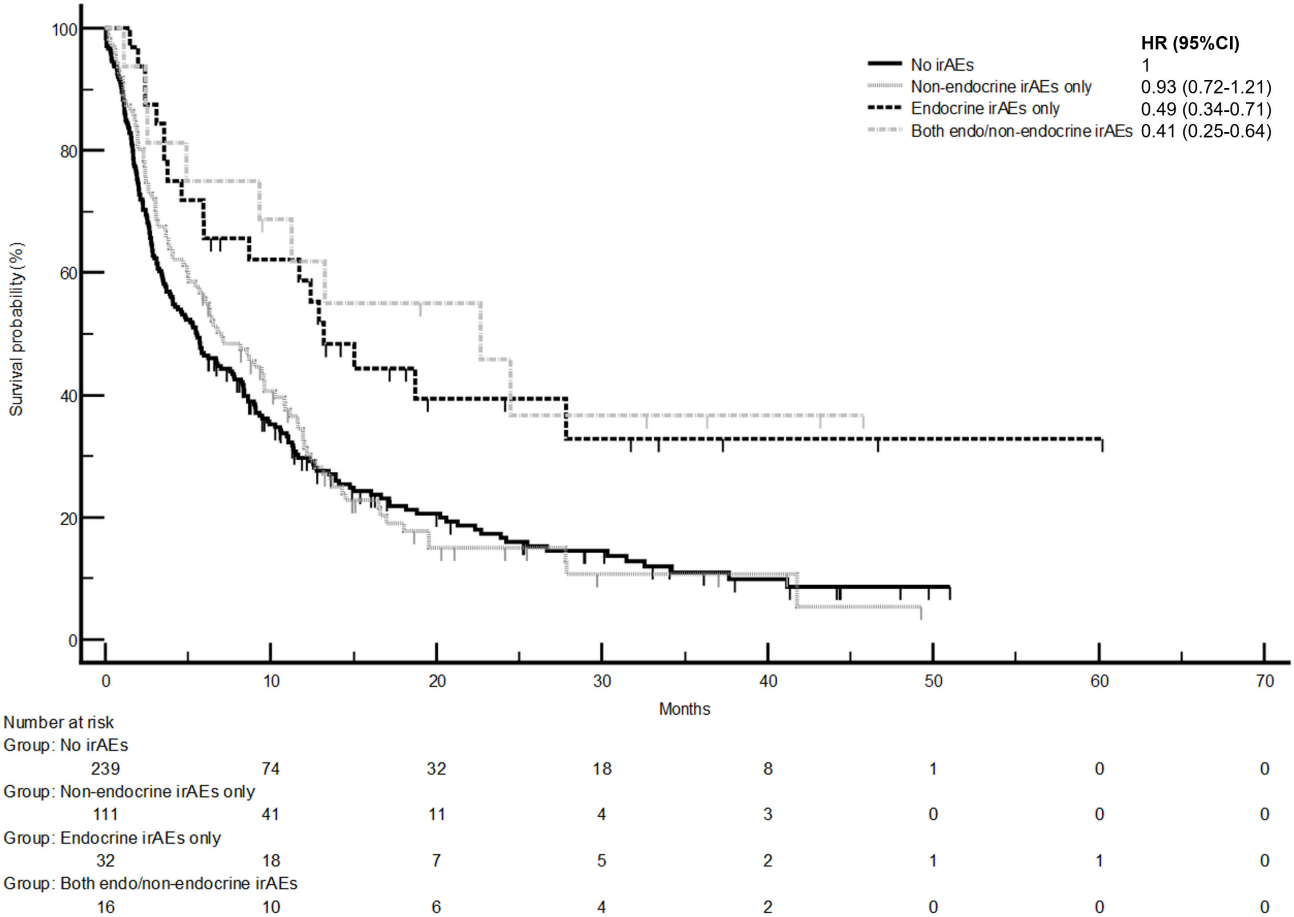
Time to treatment failure in patients with and without endocrine and/or non-endocrine immune related adverse events (irAEs). Time to treatment failure of patients who experienced non-endocrine irAEs only was 7.0 months (95%CI: 5.0-9.6, 91 events), that of patients who experienced endocrine irAEs only was 13.2 months (95%CI: 5.9-27.9, 19 events), that of patients who experienced both non-endo and endo irAEs was 22.7 months (95%CI: 4.9-24.5, 9 events). Log-rank p values = 0.001 (comparison with no irAEs for hazard ratio).

The development of non-endocrine irAEs or endocrine irAEs had positive effects on overall survival and time to treatment failure (**Figures 1** and **2**). Since the majority of endocrine irAEs in this study comprised primary thyroid dysfunction, the survival benefits of thyroid irAEs alone were similar to those observed in the endocrine irAEs group. Comparing overall survival between groups (**Figure 3**), this was significantly improved in patients who developed non-endocrine irAEs (HR 0.66, CI 0.49 – 0.89) compared with no irAEs. This was further improved in patients who developed endocrine irAEs (HR 0.54, CI 0.34 – 0.85), with the biggest survival advantage in those who developed both non-endocrine irAEs and endocrine irAEs (HR 0.16, CI 0.09-0.28). Consistent with this, time to treatment failure (**Figure 4**) was significantly improved in patients who developed endocrine irAEs (HR 0.49, CI 0.34 – 0.71) and in those who developed both non-endocrine and endocrine irAEs (HR 0.41, CI 0.25 – 0.64) but not in those who developed non-endocrine irAEs only (HR 0.93, CI 0.71 – 1.21), when compared with the no irAEs group. Overall survival according to cancer subtype is shown in **Supplementary Figure 1** (NSCLC) and **Supplementary Table 1**, showing a survival benefit in the NSCLC subgroup associated with endocrine irAEs and thyroid irAEs, but not non-endocrine irAEs. **Supplementary Table 2** shows overall survival in patients developing non-endocrine irAEs, endocrine irAEs and thyroid irAEs according to type of immune checkpoint inhibitor.

Fixed multivariable analysis including primary tumor type, age and sex (**Supplementary Table 3** and **Supplementary Table 4**), showed showed that both endocrine irAEs (HR 0.50,95%CI: 0.30-0.82) and non-endocrine irAEs (HR 0.66, 95%CI: 0.48-0.90) were significantly associated with a decreased risk of death in comparison to the non-irAEs group, whilst only endocrine irAEs were confirmed to be associated with a decreased risk of treatment failure (HR 0.48, 95%CI: 0.32-0.73).

After adjustment for time/exposure, significance was lost for the risk of death/treatment failure for both non-endocrine irAEs and endocrine irAEs groups, with only endocrine irAEs maintaining a trend towards improved survival outcomes (**Supplementary Table 1** and **Supplementary Table 2**).

## Discussion

This is the first single-center, real-world study describing the occurrence of endocrine and non-endocrine irAEs following use of CTLA4, PD-1 and PD-L1 inhibitors in a broad range of solid tumor malignancies and to investigate their association with cancer survival.

Consistent with previous findings, we report a 12% incidence of endocrine irAEs across all solid tumor malignancies treated with CTLA4, PD-1 and PD-L1 inhibitors in our cohort (5, 7, 23). Primary thyroid dysfunction was the most common endocrine irAE, accounting for 79% of all endocrine irAE cases and particularly associated with the anti-PD-1 inhibitor pembrolizumab, which is in agreement with previous studies (5, 7). Biphasic thyroiditis, a T lymphocyte mediated process, was the most frequently observed thyroid dysfunction in our study and is most commonly associated with anti PD-1 and anti-PDL1 treatment (24, 25). Previous studies have specifically identified that thyroiditis following anti-PD1 blockade may confer survival benefit in patients with advanced non-small-cell lung cancer (NSCLC) (26, 27). In accordance with this, NSCLC was the largest cancer subgroup in our study and improved overall survival was observed in NSCLC patients who developed thyroid irAEs.

Immune-related hypophysitis is most frequently associated with the CTLA-4 inhibitor ipilimumab and although hypophysitis has also been reported in patients receiving anti-PD1/PD-L1 therapy, this occurs with a much lower incidence (28). Interestingly, hypophysitis secondary to ipilimumab either alone or in combination with nivolumab has a distinct clinical phenotype to hypophysitis induced by anti-PD1 monotherapy (28–32). Hypophysitis associated with ipilimumab-based regimens typically presents with headache, loss of multiple anterior pituitary hormone axes, most frequently ACTH deficiency, and pituitary enlargement on MRI. Animal studies have found the pituitary gland expresses CTLA-4 in a subset of cells, which are targets for CTLA-4 antibody binding and this may explain the predisposition towards hypophysitis with anti-CTLA-4 therapy (33). In contrast, PD-1 and PD-L1 inhibitors often cause an isolated ACTH deficiency without headache or radiological changes. In our study, only two patients with ICI-associated hypophysitis presented with headache, multiple anterior pituitary hormone deficiencies and abnormalities on imaging, both of which had received anti-CTLA-4 inhibitors as combination therapy.

There were no reported incidences of ICI-associated primary adrenal insufficiency in our cohort. This is consistent with this being described as a rare complication of ICI treatment (32, 34).

DM developed much later than other endocrine irAEs in this study (median onset 36 weeks) and all cases occurred with the anti-PD-1 inhibitor pembrolizumab, which is in agreement with previously published data (22). DM is a less common endocrine irAE associated with ICI treatment, with a reported incidence of 0.2-1.4% (3). Consistent with this, the incidence of DM was only 0.8% in our population (22). All three patients presented with diabetic ketoacidosis and had low C-peptide levels, however, only one patient had positive anti-GAD and anti-islet cell autoantibodies. Approximately 25% of ICI-treated patients who do not develop DM show positive anti-GAD antibodies (35). ICI-induced DM can be categorised into early-onset and late-onset disease, with higher rates of anti-GAD positivity seen in early-onset DM (36). All the cases of DM in our study were late onset (range 9 – 48 weeks).

Exacerbation of pre-existing autoimmune disease is a well-recognised phenomenon of ICI inhibitor treatment, and this is often an exclusion criteria for clinical trials of ICI therapy (37, 38). However, pre-existing autoimmune disease was not an exclusion criterion for ICI therapy within the standard clinical practice of our center. In our cohort, 14.8% of patients had pre-existing autoimmune disease. There was no statistical difference between those developing non-endocrine irAEs, endocrine irAEs and those with no irAEs, which is comparable with previous reports (39). There was a significantly higher proportion of women who developed endocrine irAEs (56.2%) compared to the overall study population (37.9% female). This likely reflects the higher overall incidence of endocrinopathies in women (40), yet there is currently no consensus regarding whether there are any sex differences in immune responses to ICI treatment (41–43). Notably, the majority of endocrine irAEs reported in the current study are related to primary thyroid dysfunction and women have an increased baseline risk of autoimmune thyroid disease (44). Interestingly, a large meta-analysis of the sex differences in the risk of developing irAEs following ICI treatment showed an increased risk of symptomatic irAEs in women, but not specifically endocrine irAEs (45). Future studies are needed focusing on delineating the relationship between subtype of endocrine irAEs and sex.

Our current clinical protocol does not include measuring thyroid autoantibodies prior to ICI initiation. This is a limitation that a future, prospective study could address. Anti-thyroid peroxidase and anti-thyroglobulin antibodies may predict the development of thyroid irAEs with ICI treatment (46). Furthermore, the risk of developing non-thyroid endocrine irAEs such as hypophysitis, may be increased in patients who are positive for anti-thyroid peroxidase antibodies at baseline (21, 47). Pre-existing anti thyroid peroxidase antibodies are associated with improved prognosis in patients with advanced NSCLC receiving the PD-1 inhibitors nivolumab or pembrolizumab (48). Therefore, future, prospective studies including baseline thyroid autoantibody status would be important to further investigate associations between autoimmunity and endocrine irAEs following ICI treatment.

This study is limited by its retrospective observational design. We have not recorded data regarding whether ICIs were discontinued according to the development of irAEs. Such suspension or discontinuation of ICI treatment may shorten exposure to treatment, which may impact survival outcomes and may reduce the likelihood of patients reporting more than one ICI associated irAE. Similarly, we did not record details of the specific stage and grade of disease, histological features, location and number of metastases, and ECOG Performance Status Scale, all which may impact on cancer survival outcomes. Furthermore, face-to-face clinic appointments were reduced during the study period due to the COVID-19 pandemic, affecting patient monitoring. Since data were collected during standard clinical care, patients only had regular biochemical monitoring for thyroid dysfunction during follow up. Other endocrine irAEs were identified following clinical assessment and subsequent investigation rather than via regular biochemical monitoring. Both these approaches are consistent with recent recommendations for management of immune-related endocrinopathies in patients receiving ICI treatment for cancer (22). Nevertheless, endocrine irAEs may have developed earlier than reported or may have been missed if transient.

There are reports of rarer endocrinopathies associated with ICI treatment, including diabetes insipidus, hypoparathyroidism and autoimmune polyglandular syndromes (9, 49). However, we focussed on endocrine irAEs most likely to be encountered in clinical practice (8, 9).

Typically, irAEs associated with ICI treatment are graded using the five point Common Terminology Criteria for Adverse Events (CTCAE) system. However, the CTCAE has limitations for reporting of endocrine irAEs (32, 50) and in our institution, we do not routinely grade endocrine irAEs according to CTCAE criteria. However, future studies of the relationship between ICI treatment, the grade of severity of endocrine and non-endocrine irAEs and survival outcomes could be interesting.

Our survival analyses are in keeping with prior evidence highlighting an association between irAEs and improved outcomes in patients receiving ICI treatment (15–17). Earlier studies investigating survival outcomes and ICI-associated irAEs have been limited to clinical trials of the effects of specific ICI treatments in patients with advanced cancers of a single tumor type (15–17). Interestingly, our findings are also consistent with recent studies of ICI-associated endocrine irAEs and their positive effects on survival in patients with metastatic solid organ malignancy of different types (21, 23). Building on work by Paschou et al., our larger study included patients treated with anti-CTLA4 inhibitors and also determined risk factors for developing endocrine irAEs (23). Although several of these prior studies included similar classes of ICI to those in our study (21), both had a shorter follow up period. The current study is the first to compare the effects of endocrine irAEs with non-endocrine irAEs on survival. Therefore, our work adds to the limited literature comprising real-world clinical data on development of irAEs and survival benefit with ICI treatment.

Many studies of the relationship between endocrine and non-endocrine irAEs associated with ICI treatment and survival are not adjusted for immortal time bias (ITB). ITB may be introduced when patients remain in a study for a shorter time period if they develop disease progression or die earlier compared to those patients who remain in the study for a longer period if they remain free of disease progression, hence potentially having an increased risk of developing a clinical outcome. Time-adjusted analyses acknowledge a change in treatment exposure status over time. In the current study, adjustment for time showed the survival benefit of endocrine irAEs was lost but the trend remained. Interestingly, a recent comprehensive meta-analysis assessing the relationship between thyroid irAEs with ICI treatment and cancer survival outcomes showed positive benefits of thyroid irAEs on overall survival persisted when adjusting for ITB (51). In contrast, the differences in survival outcome associated with the development of hypophysitis from PD-1 blockade or CLTA-4 inhibitors are less clear when adjusting for ITB (52, 53). The positive trend restricted towards endocrine irAEs indicates this class of adverse events as a potential pharmacodynamic marker of ICI activity, with possible T-cell cross-reactivity between the tumor and endocrine irAE tissues as the most likely underlying mechanistic link (54).

There is a need for further studies in this field as endocrinopathies are associated with considerable morbidity. This is especially relevant in younger patients achieving cancer remission following ICI treatment, but in whom permanent endocrine dysfunction may cause a significant negative impact on quality of life. This study and others are retrospective. Future, larger prospective studies are needed to further investigate the risk factors for irAEs, with the aim to develop a personalised approach to the selection of ICI agent(s) and surveillance strategy based on individualised risk for developing irAEs.

## Data availability statement

The data analyzed in this study is subject to the following licenses/restrictions: This is anonymised clinical data. This will not be accessible to any individuals outside of our hospital.

## Ethics statement

During the organization of this audit of nonidentifiable data from routine clinical practice, the Health Research Authority online decision tool http://www.hra-decisiontools.org.uk/research/ was used which confirmed that regulatory approval by an NHS Research Ethics Committee (REC) was not required. This study was registered as an audit with our institution (audit number END_023). The studies were conducted in accordance with the local legislation and institutional requirements. This was a retrospective, observational study of clinical data. These data were anonymized and all patient identifiable details removed. Hence, no individual patient consent was required.

## Author contributions

LY: Writing – review & editing, Writing – original draft, Investigation, Formal Analysis, Data curation. SMu: Writing – review & editing, Writing – original draft, Investigation, Data curation. AC: Validation, Software, Formal Analysis, Writing – review & editing, Writing – original draft, Data curation. EL: Methodology, Investigation, Writing – review & editing, Writing – original draft, Formal Analysis. MG: Resources, Conceptualization, Writing – review & editing, Writing – original draft. DP: Formal Analysis, Data curation, Writing – review & editing, Writing – original draft. MA: Methodology, Writing – review & editing, Writing – original draft. SMa: Resources, Writing – review & editing, Writing – original draft. NM: Visualization, Validation, Supervision, Software, Project administration, Methodology, Investigation, Funding acquisition, Formal Analysis, Data curation, Conceptualization, Writing – review & editing, Writing – original draft, Resources.
