# Supplementary material for: Effects of immune checkpoint inhibitor associated endocrinopathies on cancer survival
Source: Front Endocrinol (Lausanne). 2024 Apr 12;15:1369268. doi: 10.3389/fendo.2024.1369268 (PMC11045886; doi:10.3389/fendo.2024.1369268)
Supplement: Supplementary file 1 [file DataSheet_1.pdf]

Supplementary Table 1.

Overall survival in patients developing non-endocrine immune related adverse events (irAEs), endocrine irAEs and thyroid irAEs according to cancer subtype.

	**Risk of death HR (95%CI)**
**Melanoma (n = 42)**	
Non-endocrine irAEs vs No irAEs	1.63 (0.34-7.70)
Endocrine irAEs vs No irAEs	0.96 (0.13-6.94)
Thyroid irAEs vs No irAEs	Not computable
**Renal cell and urothelial carcinoma (n = 78)**	
Non-endocrine irAEs vs No irAEs	0.44 (0.22-0.88)
Endocrine irAEs vs No irAEs	0.35 (0.11-1.14)
Thyroid irAEs vs No irAEs	0.59 (0.18-1.95)
**Head and Neck carcinoma (n = 39)**	
Non-endocrine irAEs vs No irAEs	0.56 (0.13-2.38)
Endocrine irAEs vs No irAEs	0.72 (0.25-2.11)
Thyroid irAEs vs No irAEs	0.76 (0.25-2.11)
**Others (n = 16)**	
Non-endocrine irAEs vs No irAEs	0.23 (0.03-1.87)
Endocrine irAEs vs No irAEs	Not computable
Thyroid irAEs vs No irAEs	Not computable

Non small cell lung cancer data shown in Supplementary Figure 1. irAEs = immune related adverse events.

Supplementary Table 2.

Overall survival in patients developing non-endocrine immune related adverse events (irAEs), endocrine irAEs and thyroid irAEs according to type of immune checkpoint inhibitor.

	**Risk of death HR (95%CI)**
**PD-1/CTLA-4 inhibitors (n = 37)**	
Non-endocrine irAEs vs No irAEs	0.76 (0.27-2.15)
Endocrine irAEs vs No irAEs	0.75 (0.18-3.03)
Thyroid irAEs vs No irAEs	0.54 (0.07-4.62)
**PD-1 inhibitors (n = 258)**	
Non-endocrine irAEs vs No irAEs	0.51 (0.35-0.77)
Endocrine irAEs vs No irAEs	0.32 (0.17-0.63)
Thyroid irAEs vs No irAEs	0.33 (0.16-0.68)
**PD-L1 inhibitors (n = 103)**	
Non-endocrine irAEs vs No irAEs	0.74 (0.43-1.29)
Endocrine irAEs vs No irAEs	0.53 (0.21-1.35)
Thyroid irAEs vs No irAEs	0.53 (0.21-1.35)

Combination anti-PD-1 and anti-CTLA4 inhibitors: nivolumab plus ipilimumab (n = 37).

Anti-PD-1 inhibitors: nivolumab (n = 57) or pembrolizumab (n = 201)

Anti-PD-L1 inhibitors: ateluzumab (n = 85), durvalumab (n = 6) or avelumab (n = 12).

Supplementary Table 3

Multivariable analyses for risk of death and time-adjusted risk of death in patients developing non-endocrine immune related adverse events (irAEs) compared with no irAEs

	**Risk of death HR (95%CI)**	**Time-adjusted risk of death HR (95%CI)**
**Non-Endocrine irAEs**	0.66 (0.48-0.90)	1.08 (0.78-1.49)
**Age**		
Median (range)	0.99 (0.98-1.01)	0.99 (0.98-1.01)
**Sex**		
Male vs Female	1.41 (1.04-1.89)	1.40 (1.05-1.08)
**Primary Tumour**		
NSCLC	1	1
Melanoma	0.39 (0.20-0.77)	0.32 (0.17-0.61)
Renal Cell Carcinoma	0.85 (0.54-1.33)	0.82 (0.53-1.26)
Urothelial Carcinoma	1.74 (1.09-2.78)	1.85 (1.19-3.05)
HCC	0.77 (0.33-1.79)	0.76 (0.33-1.74)
Head and Neck Cancers	1.59 (1.03-2.47)	1.72 (1.14-2.62)
Others	0.37 (0.11-1.21)	0.34 (0.11-1.09)
	**Risk of treatment failure HR (95%CI)**	**Time-adjusted risk of treatment failure HR (95%CI)**
**Non-Endocrine irAEs**	0.83 (0.64-1.07)	1.34 (1.01-1.76)
**Age**		
Median (range)	0.99 (0.98-1.01)	0.99 (0.98-1.01)
**Sex**		
Male vs Female	1.27 (0.98-1.63)	1.35 (1.06-1.71)
**Primary Tumour**		
NSCLC	1	1
Melanoma	0.74 (0.47-1.18)	0.65 (0.42-0.99)
Renal Cell Carcinoma	2.53 (1.76-3.65)	2.15 (1.52-3.03)
Urothelial Carcinoma	1.38 (0.89-2.15)	1.48 (0.95-2.28)
HCC	1.18 (0.62-2.27)	1.18 (0.62-2.25)
Head and Neck Cancers	1.49 (1.01-2.22)	1.62 (1.13-2.40)
Others	0.95 (0.48-1.90)	0.95 (0.50-1.81)

Time-adjusted Cox multivariable regression to estimate the risk of death and of treatment failure for non-endocrine irAEs vs no irAEs. NSCLC = non small cell lung cancer. HCC = hepatocellular carcinoma.

Supplementary Table 4

Multivariable analyses for risk of death and time-adjusted risk of death in patients developing endocrine immune related adverse events (irAEs) compared with no irAEs

	**Risk of death**<br>**HR (95%CI)**	**Time-adjusted risk of death**<br>**HR (95%CI)**
**Endocrine irAEs**	0.50 (0.30-0.82)	0.78 (0.47-1.27)
**Age**		
Median (range)	0.99 (0.98-1.01)	0.99 (0.98-1.01)
**Sex**		
Male vs Female	1.42 (1.02-1.96)	1.39 (1.04-1.84)
**Primary Tumour**		
Lung	1	1
Melanoma	0.31 (0.11-0.85)	0.34 (0.18-0.63)
Renal Cell Carcinoma	0.94 (0.55-1.58)	0.83 (0.54-1.27)
Urothelial Carcinoma	1.96 (1.19-3.22)	1.87 (1.18-2.96)
HCC	1.09 (0.43-2.73)	0.75 (0.33-1.73)
Head and Neck Cancers	1.75 (1.13-2.70)	1.72 (1.13-2.60)
Others	0.37 (0.11-1.21)	0.34 (0.11-1.10)
	**Risk of treatment failure**<br>**HR (95%CI)**	**Time-adjusted risk of treatment failure**<br>**HR (95%CI)**
**Endocrine irAEs**	0.48 (0.32-0.73)	0.87 (0.57-1.33)
**Age**		
Median (range)	0.99 (0.98-1.01)	0.99 (0.98-1.01)
**Sex**		
Male vs Female	1.25 (0.94-1.67)	1.36 (1.07-1.73)
**Primary Tumour**		
Lung	1	1
Melanoma	0.56 (0.28-1.11)	0.69 (0.45-1.07)
Renal Cell Carcinoma	3.25 (2.10-5.03)	2.28 (1.62-3.19)
Urothelial Carcinoma	1.58 (0.99-2.52)	1.45 (0.94-2.24)
HCC	1.25 (0.54-2.89)	1.17 (0.61-2.23)
Head and Neck Cancers	1.64 (1.10-2.45)	1.60 (1.10-2.32)
Others	0.93 (0.45-1.94)	0.94 (0.49-1.78)

Time-adjusted Cox multivariable regression to estimate the risk of death and of treatment failure for endocrine irAEs vs no irAEs.
