# Supplementary material for: Effects of immune checkpoint inhibitor associated endocrinopathies on cancer survival
Source: Front Endocrinol (Lausanne). 2024 Apr 12;15:1369268. doi: 10.3389/fendo.2024.1369268 (PMC11045886; doi:10.3389/fendo.2024.1369268)
Supplement: Supplementary file 2 [file DataSheet_2.pdf]

Supplementary Figure 1

Overall survival in patients with non small cell lung cancer (NSCLC)

N = 209 patients.

A Overall survival for non-endocrine immune related adverse events (irAEs) was 16.6 months (95%CI: 11.4-38.3; 38 events) vs no irAEs: 12.6 months (95%CI: 9.2-16.5; 86 events), p=0.1682.

B Overall survival for endocrine irAEs: 38.3 months (95%CI: 19.4-38.3; 9 events) vs no irAEs: 12.6 months (95%CI: 9.2-16.5; 86 events), p=0.0061.

C Overall survival for thyroid irAEs: not reached (7 events) vs no irAEs: 12.6 months (95%CI: 9.2-16.5; 86 events), p=0.0028.
