# Supplementary figures and images for: Effects of immune checkpoint inhibitor associated endocrinopathies on cancer survival
Source: Front Endocrinol (Lausanne). 2024 Apr 12;15:1369268. doi: 10.3389/fendo.2024.1369268 (PMC11045886; doi:10.3389/fendo.2024.1369268)

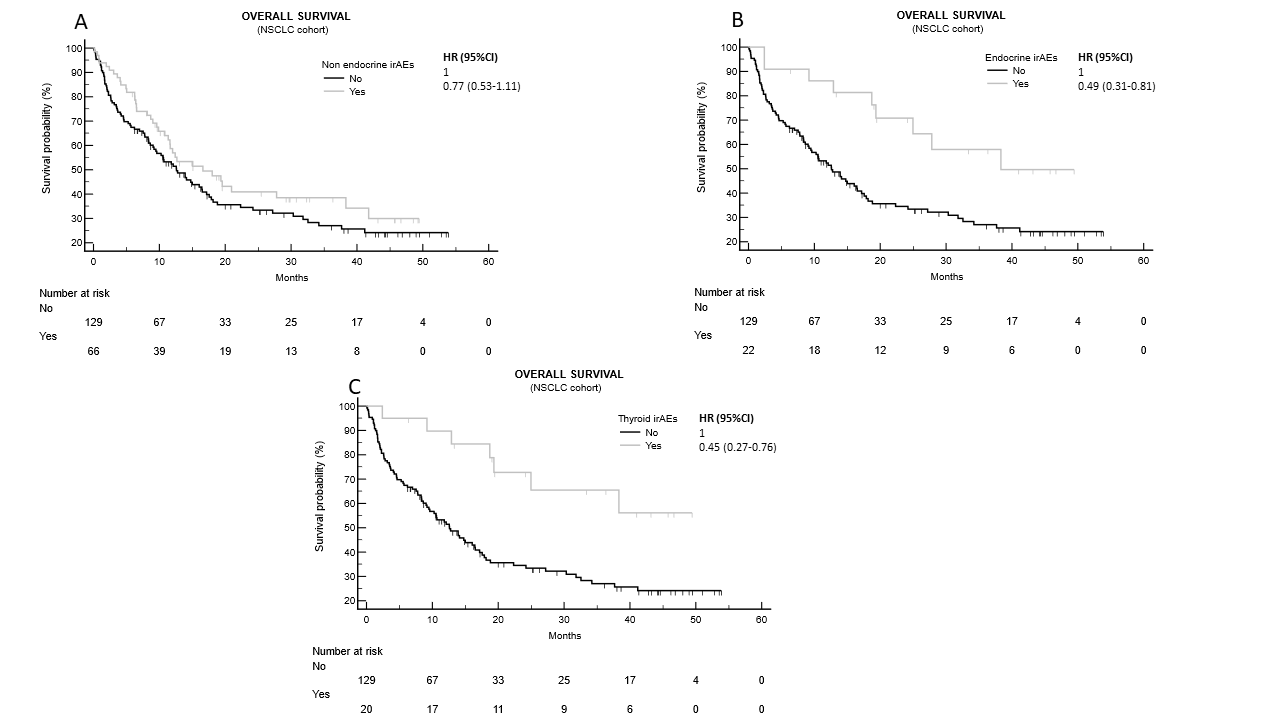

Supplement: Supplementary file 3 [file Image_1.tif]
